# Hybridization for human epidermal growth factor receptor 2 testing in gastric carcinoma: a comparison of fluorescence *in-situ* hybridization with a novel fully automated dual-colour silver *in-situ* hybridization method

**DOI:** 10.1111/j.1365-2559.2011.03894.x

**Published:** 2011-07

**Authors:** Elena García-García, Carlos Gómez-Martín, Bárbara Angulo, Esther Conde, Ana Suárez-Gauthier, Magdalena Adrados, Cristian Perna, José Luis Rodríguez-Peralto, Manuel Hidalgo, Fernando López-Ríos

**Affiliations:** Laboratorio de Dianas Terapéuticas, Centro Integral Oncológico ‘Clara Campal’, Hospital Universitario Madrid Sanchinarro, Universidad San Pablo-CEUFacultad de Medicina; 1 Gastrointestinal Cancer Clinical Research Unit, Clinical Research Program, Spanish National Cancer Research Centre (CNIO)Madrid; 2 Hospital Universitario de la PrincesaMadrid; 3 Hospital Universitario 12 de Octubre

**Keywords:** dual-colour hybridization, fluorescence *in-situ* hybridization, gastric carcinoma, human epidermal growth factor receptor 2, silver-enhanced *in-situ* hybridization

## Abstract

**Aims:**

Amplification of the human epidermal growth factor receptor 2 (*HER2*) gene has been reported in gastric carcinoma (GC). Accordingly, trastuzumab plus chemotherapy has recently become the new standard treatment for HER2-positive advanced GCs. The aim was to compare the alleged gold standard for hybridization [fluorescence *in-situ* hybridization (FISH)] with a novel, fully automated brightfield dual-colour silver-enhanced *in-situ* hybridization (SISH) method.

**Methods and results:**

The studies series was comprised of 166 GC samples. Additionally, tumours with discordant results obtained by FISH and SISH were analysed by real-time quantitative polymerase chain reaction (PCR) with the LightMix kit HER-2/*neu*. Of the samples, 17.5% and 21% were amplified by FISH and SISH, respectively. Heterogeneity was identified in up to 52% of cases. In 96.4% of cases, FISH showed the same results as SISH. All six discordant cases were positive by SISH and negative by FISH. On review of the FISH slides, all contradictory cases were polysomic and were confirmed to be negative for amplification by real-time PCR. Interestingly, all ratios in this latter group were between 2.06 and 2.50, so setting the cut-off for amplification at ≥3 resulted in perfect concordance.

**Conclusions:**

Dual-colour SISH represents a novel method for the determination of *HER2* status in GC.

## Introduction

In 1987, Slamon *et al.* described the relationship between the amplification of the human epidermal growth factor receptor 2 (*HER2*) gene and a group of breast carcinomas^1^ for the first time. The development, in later years, of a specific treatment for this alteration, the antibody trastuzumab, has been one of the greatest successes of solid tumour oncology. Naturally, there have been attempts to reproduce this success in other neoplasias that can also show amplification of *HER2* (carcinomas of the stomach, bladder, lung etc.).^2^–^4^ Finally, in the year 2009, results were presented of the ToGA trial in patients with advanced gastric carcinoma (GC).^2^

The ToGA trial was a prospective, randomized, multicentre phase III trial conducted in 24 centres. This study looked for *HER2* expression/amplification in 3807 patients with GCs, and found a positivity rate of 22% [either by fluorescence *in-situ* hybridization (FISH) or immunohistochemistry (IHC) 3+]. Five hundred and eighty-four HER2-positive GCs were included and randomized between cisplatin plus fluoropyrimidine (capecitabine) (Ch) or the same chemotherapy plus trastuzumab (Ch+T). Patients treated with Ch+T showed a clinically and statistically significant improvement in terms of overall survival (13.8 months versus 11.1 months, hazard ratio 0.7, 95% confidence interval 0.6–0.9, *P* = 0.0046). Secondary endpoints of the study were also met; thus Ch+T significantly improved the overall response rate and progression-free survival, as compared with Ch alone, without any increase in toxic effects, including cardiac events. Therefore, Ch+T chemotherapy has become the new standard treatment for HER2-positive advanced gastric cancer.^2^

However, since trastuzumab was first approved for breast cancer, there have been notable inconsistencies in procedures for studying HER2. This ‘cancer biomarker problem’ significantly affected the specific method: IHC, FISH or the various types of brightfield *in-situ* hybridization (ISH). Similarly, it affected the concordance between techniques; the cut-off point and the workflow algorithm although IHC has traditionally been considered to be the primary testing modality.^5^–^7^ The facts that the first American Society of Clinical Oncology/College of American Pathologists (CAP) consensus was not published until 2007^8^ and that there is a lack of consensus among different national guidelines (reviewed in Bilous *et al.*^9^) undoubtedly contributed to the problem. Further fuelling the controversy was the subtle recommendation of initial FISH in specific settings (core-needle biopsies)^10^ and the direct suggestion of ‘FISH as the primary HER2 testing modality for women with breast cancer who are candidates for HER2-targeted therapies’.^11^ This latter paradigm shift has remained largely unnoticed, despite being published in a high-impact journal and the fact that Slamon was among its authors more than 20 years after his initial discovery. The situation that we have outlined above has recently been termed ‘the HER2 testing conundrum’, and the result is that as many as one in five HER2 tests give the wrong answer.^12^,^13^

However, it must be pointed out that the clinical benefit of trastuzumab in the ToGA trial seems to be restricted to IHC 2 + and FISH-positive or IHC 3 + patients, in the latter situation irrespective of the FISH status.^2^

With the above controversies in mind, we sought to compare the alleged gold standard (FISH) with a novel fully automated brightfield dual-colour approach in a series of GC samples.^14^ Our aim was to provide robust analytical and post-analytical information in the setting of GC to guide the clinical validation of the different assays, as has been suggested.^15^

## Materials and methods

### Tumour samples

A randomly selected total of 166 gastric adenocarcinomas from several Spanish institutions were collected for HER2 status analyses. The study was approved by a centralized ethics committee. Before paraffin embedding, tissues were fixed in buffered formalin. We have no data regarding how concentrated the fixative was or the duration of fixation. Fifty (30.1%) were endoscopic biopsy specimens and 116 (69.9%) were surgical specimens, of which 15 (9%) were distant metastases. All cases were diagnosed according to Lauren's classification:^16^ 86 (51.8%) intestinal type, 47 (28.3%) diffuse type and 33 (19.9%) indeterminate type.

### Analytical phase

Sections of tumour tissue samples 4 μm thick were cut and placed on charged polylysine-coated slides for analysis.

### FISH

*HER2* copy number was investigated by FISH, using the PathVysion *HER2* DNA probe kit (Vysis, Downers Grove, IL, USA), with the Dako Histology FISH Accessory kit. For the Dako Histology kit, the manufacturer's instructions were modified, in order to optimize the technique (decreased laboratory processing).^17^ Sections were incubated at 56°C overnight, deparaffinized in two series of xylol, and rehydrated with an ethanol series. Slides were pretreated with Pre-treatment Solution in a water bath at 97°C for 10 min. Enzymatic digestion was carried out with Ready-to-Use Pepsin for 3 min at room temperature (endoscopic biopsies) or 6 min at 37°C (surgical specimens). After dehydration with a graded ethanol series, 10 μl of *HER2*/*CEP17* probe mix was applied to each tissue section. The slides and probe were denatured at 80°C for 5 min and hybridized at 37°C overnight in a Dako Hybridizer. On the second day, the sections were washed with Stringent Wash Buffer at 65°C for 10 min in a water bath. Then, the slides were dehydrated with a graded ethanol series, and 10 μl of fluorescence mounting medium containing 4′,6-diamino-2-phenylindole (DAPI) was applied.

### Dual-colour silver-enhanced *in-situ* hybridization (SISH)

Automated SISH was performed on a Ventana Benchmark XT (Ventana Medical Systems, Tucson, AZ, USA). INFORM *HER2* DNA Probe and INFORM Chromosome 17 Probe were visualized on the same slide, following the manufacturer's protocols, with a few variations. Assay conditions were modified for optimal results. The entire assay procedure (deparaffinization, pretreatment, hybridization, stringency wash, signal detection and counterstaining) was fully automated.^14^ Pretreatment was performed with Reaction Buffer and enzyme digestion with ISH Protease 3 for 12 min. *HER2* probe was denatured at 95°C for 15 min and hybridized at 56 C for 6 h. Chromosome 17 centromere probe was denatured at 95 C for 12 min and hybridized at 44°C for 3 h. Stringency washes for *HER2* probe were performed at 72°C for 8 min (three steps) and incubated with anti-dinitrophenol (DNP) antibody for 20 min. Then, tissue sections were incubated with horseradish peroxidase-conjugated anti-rabbit antibody for 16 min. The silver signal for *HER2* was revealed by sequential silver reactions (Silver C incubation time, 4 min). For chromosomal 17 centromere probe, three stringency washes were performed at 59°C for 8 min. Then, tissue sections were incubated with anti-DNP antibody for 20 min and with alkaline phosphatase-conjugated antibody for 12 min. The signal of the centromere was visualized with the Red ISH Naphthol reaction for 4 min. The tissues were counterstained with Hematoxylin II for 8 min and Bluing Reagent for 4 min. The slides were covered with Cytoseal mounting medium. Some of the slides had to be stained twice. We were not able to identify the cause of these failures, which other authors have also experienced.^18^

### Real-time polymerase chain reaction (PCR)

Although FISH is still considered to be the gold standard for *HER2* amplification in the clinical setting, we sought to study our discordant cases (see below) by a third technique.^19^ Real-time quantitative PCR was performed with the LightMix kit HER-2/*neu* (Roche Diagnostics, Indianapolis, IN, USA). A 101-bp fragment of *HER2* and a 119-bp fragment of the *RPL23* reference gene, both localized on chromosome 17q21, were amplified according to the manufacturer's instructions. Simultaneous quantification of *HER2* and of the reference gene was accomplished by using two different LightCycler hybridization probes (LightCycler Red 640 and LightCycler Red 670, respectively), enabling dual-colour detection in a single test tube. A colour compensation file generated with the Roche Diagnostics LightCycler Multicolour Compensation Set was used to correct the fluorescence in the duplex reaction. DNA was extracted from formalin-fixed paraffin-embedded tumour tissue with a previously described protocol,^20^ and subsequently amplified in triplicate with a LightCycler 480 real-time PCR instrument (Roche Diagnostics). In each PCR experiment, DNA extracted from tumours with known *HER2* amplification status (tumours classified as amplified or non-amplified by both FISH and SISH) was included as a positive control: three samples without amplification and three samples with amplification. Moreover, each PCR experiment included a non-template control and standards (from 10^1^ to 10^6^ equivalents per reaction of *HER2* DNA and of reference DNA) supplied with the kit. These standards allow the generation of standard curves for both products to determine the linear range of both PCR reactions and to estimate the quantity of the target sequence in unknown samples. Briefly, for each reaction, 2 μl of LightCycler *HER2* mix, 2 μl of LightCycler reference mix, 10 μl of LightCycler 480 probes master mix and 1 μl of PCR-grade water were combined. Five microlitres of DNA (for samples and standards) or PCR-grade water (for negative control) was added, to give a total volume of 20 μl. PCR was performed as follows: after an initial 10 min denaturation of DNA at 95°C, 45 amplification cycles were performed. Each cycle consisted of denaturation at 95°C for 10 s, annealing at 60°C for 10 s, and extension at 72°C for 10 s. The fluorescence signals were measured after each annealing step.

### Post-analytical phase (interpretation)

The slides were analysed by two observers. All of the preparation was previously evaluated (×10 and ×40 objectives in the case of SISH, and ×100 objective in the case of FISH) to identify areas for scoring and to avoid bias resulting from tumour heterogeneity.

For FISH, 20 nuclei were scored from two different areas, using an epifluorescence microscope (Olympus BX61) equipped with a DAPI/Spectrum Orange/Spectrum Green double-filter set, using a × 100 oil immersion objective lens. The scoring of SISH was similarly conducted with the use of a brightfield microscope (Olympus BX41) with a × 40 objective. *HER2* amplification was considered to be positive when the ISH ratio was ≥2, and negative when the ISH ratio was <2. Chromosome 17 polysomy was defined as ≥3 CEP17 signals on average per cell.^21^ Amplification patterns in clusters versus double minutes were considered according to published criteria.^22^

For data analysis in the real-time assay, the LightCycler 480 Relative Quantification software (version 1.5) provided by Roche Diagnostics (Indianapolis, IN, USA) was used. The second derivative maximum method to calculate the value of the crossing point for target and reference genes of each sample was used. The *HER2* copy number was calculated automatically as the ratio between *HER2* and the reference gene. The ratio *HER2*/reference for each sample was normalized to one of the non-amplified tumours (determined either by FISH or SISH) included as positive controls in the PCR experiment. According to the manufacturer, a ratio between *HER2* and the reference gene of <2 is regarded as negative for *HER2* amplification, whereas a ratio of ≥2 indicates amplification of *HER2*.

### Statistical analysis

The agreement between FISH and SISH was estimated by the percentage of agreement and by kappa statistics. A one-sample *Z*-test was performed in order to test the proportion of the two histological subtypes (intestinal versus diffuse and indeterminate).

## Results

### FISH

**Figure 1:**
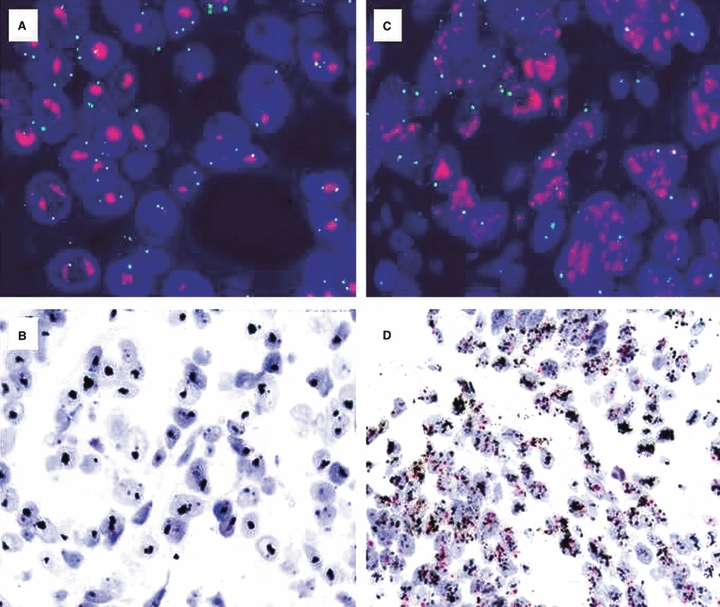
Gastric carcinoma with *HER2* amplification. **A**,**B**, amplification in clusters by (**A**) fluorescence *in-situ* hybridization (FISH) and (**B**) silver-enhanced *in-situ* hybridization (SISH). **C**,**D**, amplification in a double minute pattern by (**C**) FISH and (**D**) SISH.

FISH was successfully performed on all samples. The quality of the hybridization was good (Figure 1); 17.5% were amplified, 55% had double minute amplification and 45% had cluster amplification. Heterogeneity (focal amplification) was observed in 52% of the amplified cases. Interobserver agreement was almost perfect. For one observer, the median of the ratios was 5.25 and the range was 13.26. For the other observer, the median of the ratios was 5 and the range was 17.9. *HER2* amplification was significantly associated with the intestinal histological subtype when compared with the other categories of the Lauren classification (86% versus 14%, *P* < 0.0001).

### Dual-colour SISH

**Figure 2:**
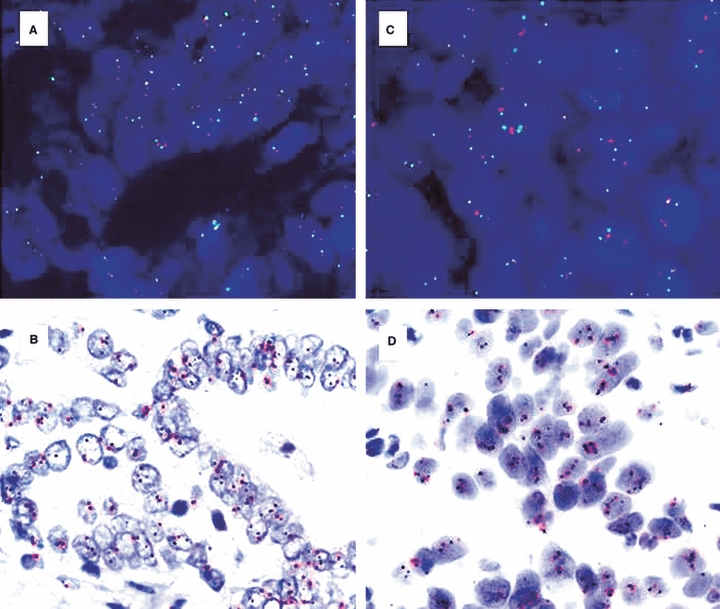
*In-situ* hybridization of *HER2* in gastric carcinoma. **A**,**B** Non-amplified case by (**A**) fluorescence *in-situ* hybridization (FISH) and (**B**) silver-enhanced *in-situ* hybridization (SISH). **C**,**D**, Chromosome 17 polysomy by (**C**) FISH and (**D**) SISH.

SISH was successfully performed on all samples. The quality of the hybridization was good (Figure 2); 21% were amplified, 34% had double minute amplification, 46% had cluster amplification and 20% had a mixed amplification pattern. Heterogeneity (focal amplification) was observed in 29% of the amplified cases. Interobserver agreement was almost perfect. For one observer, the median of the ratios was 5.75 and the range was 14.64. For the other observer, the median of the ratios was 5.65 and the range was 9.01.

### Correlation between FISH and SISH

In 96.4% of cases, FISH showed the same results as SISH. All six discordant cases were positive by SISH and negative by FISH (sensitivity of 1, specificity of 0.956, concordance of 0.964, κ = 0.884; Table 1). Upon review of the FISH slides, all contradictory cases were polysomic and were confirmed to be negative for amplification by real-time PCR (see below). Interestingly, all ratios in this latter group (including those of the two observers) were between 2.06 and 2.50, so setting the cut-off for amplification at ≥3 would result in perfect concordance (sensitivity of 1, specificity of 1, concordance of 1, κ = 1; Table 2). All of the ratios (including those of the two observers) of the SISH concordant cases were greater than 3 (medians of 8.1 and 6.02; ranges of 13.66 and 8.02).

**Table 1:** Performance of silver-enhanced *in-situ* hybridization (SISH) with a cut-off ≥2

	FISH		
		
	Positive	Negative	Total
SISH			
Positive	29	6	35

Negative	0	131	131

Total	29	137	166

FISH, fluorescence *in-situ* hybridization.

**Table 2:** Performance of silver-enhanced *in-situ* hybridization (SISH) with a cut-off ≥3

	FISH		
		
	Positive	Negative	Total
SISH			
Positive	29	0	29

Negative	0	137	137

Total	29	137	166

FISH, fluorescence *in-situ* hybridization.

### Real-time PCR

Six tumours with discordant results obtained by FISH and SISH were analysed by real-time quantitative PCR with the LightMix kit HER-2/*neu* (Roche Diagnostics), in order to classify them as amplified or non-amplified for *HER2* (Table 3). Samples amplified by FISH and SISH, considered as positive controls for *HER2* amplification, were all found to be amplified with the real-time quantitative approach. Similarly, samples assessed as negative for *HER2* amplification by both FISH and SISH gave a normalized *HER2*/*RPL23* ratio of ∼1 (range from 0.836 to 1.0), which is below the cut-off limit of 2 and confirms the absence of amplification. None of the samples with discordant results showed amplification of *HER2* by real-time quantitative PCR. After normalization of the target/reference gene ratio for each sample, values of <2 were found for all tumours, confirming the results obtained by FISH.

**Table 3:** Correlation of *HER2* status studied by fluorescence *in-situ* hybridization (FISH), silver-enhanced *in-situ* hybridization (SISH) and real-time quantitative polymerase chain reaction (PCR)

			Real-time quantitative PCR		
			
Sample ID*	FISH analysis	SISH analysis	*HER2*/*RPL23* ratio	Normalized ratio	Status
12	Non-amplified	Non-amplified	0.9272	1.0	Non-amplified

9	Non-amplified	Non-amplified	0.7752	0.8361	Non-amplified

11	Non-amplified	Non-amplified	0.8314	0.8967	Non-amplified

35	Amplified	Amplified	18.70	20.17	Amplified

84	Amplified	Amplified	12.43	13.40	Amplified

85	Amplified	Amplified	1.95	2.103	Amplified

68	Non-amplified	Amplified	0.3276	0.3533	Non-amplified

39	Non-amplified	Amplified	0.8597	0.9271	Non-amplified

67	Non-amplified	Amplified	0.8880	0.9577	Non-amplified

113	Non-amplified	Amplified	0.7460	0.8045	Non-amplified

129	Non-amplified	Amplified	0.5388	0.5811	Non-amplified

8	Non-amplified	Amplified	0.8503	0.9171	Non-amplified

* Negative controls: samples 12, 9 and 11. Positive controls: samples 35, 84 and 85. Discordant cases: samples 68, 39, 67, 113, 129 and 8.

## Discussion

In the present study, we have compared for the first time a novel, fully automated dual-colour SISH method with FISH for the assessment of *HER2* amplification in a large series of GC samples. There follows a discussion of the different phases of the procedures.

First, ISH is not affected by the unavoidable variability of the pre-analytical phase in pathology laboratories worldwide, as long as buffered formalin is used as the fixative. In our experience as a referral laboratory for FISH *HER2* testing, only 3% are considered to be non-informative because of pre-analytical aspects (F. López-Ríos, unpublished data). Expanded decalcification protocols even permit ISH in bone marrow biopsy specimens (E. García-García, unpublished data). Although the role of the new rapid fixatives remains to be determined, it must be emphasized that alcohol-based fixation is not appropriate for ISH procedures. In the present series, in spite of the fact that samples of different sizes (endoscopic versus surgical specimens, etc.) and sources (community hospitals versus large university hospitals, etc.) were studied, both methods showed very low failure rates (data not shown). These results are consistent with previous reports on failure rates for epidermal growth factor receptor (EGFR) SISH versus FISH.^23^

Second, manual ISH (i.e. FISH) remains the gold standard in this setting, and will remain so until proven otherwise. Nevertheless, new automated ISH alternatives may improve the reproducibility of the analytical phase if the technical platforms, reagents and protocols are fully standardized. In the past, there were two main limitations to the widespread use of ISH techniques: (i) FISH has traditionally been perfomed in central or referral laboratories, with a lack of community acceptance or experience; and (ii) bright-field ISH has not, until recently, been a dual-colour procedure.^14^,^24^,^25^ The new, fully automated, dual-colour SISH overcomes both limitations, but appropriate training is essential for adequate performance of the post-analytical phase (interpretation) of the procedure. This is somewhat easier and quicker for bright-field ISH than for FISH.^26^,^27^ It is beyond the scope of this study to establish the predictive value of the different ISH modalities in GC patients who are candidates for HER2-targeted therapies, but the use of automated ISH techniques may enable rapid screening of thousands of patients in order to determine the real predictive cut-off for clinical benefit (i.e. polysomy, degree of *HER2* amplification).

Discrepancy between FISH and SISH occurred in six of 166 cases, all of them positive for SISH (all ratios between 2.06 and 2.50) and negative for FISH. All discrepant cases were polysomic by FISH and negative for *HER2* amplification by real-time PCR. These results are consistent with previous studies reporting on both *EGF*R and *HER*2 brightfield ISH, both using single-colour^18^,^27^–^32^ and dual-colour^14^,^24^,^26^,^33^ approaches. However, it must pointed out that, in some series, the brightfield ISH results are ‘false’ negatives, not ‘false’ positives, and this has potential clinical consequences.^14^,^24^,^33^ Interestingly, polysomy has been reported as the major cause of response to trastuzumab in FISH-negative breast carcinoma patients.^34^ In our series, no cases showing gene amplification by FISH were considered to be negative by SISH. When we raised the SISH cut-off for amplification (≥3), the concordance was 100%, demonstrating that SISH is a valid testing method for *HER2* testing in GC patients and that a higher cut-off (≥3) should be considered even for dual-colour SISH, in order to increase the concordance rate. It is also necessary to take into account that polysomy may be the cause of inter-observer differences.^29^ A very recent report on a study using a similar methodogy to the one reported herein (fully automated SISH assay with single-colour locus detection on two separate slides) has arrived at the same conclusions: chromosome 17 counts are the main source of discrepancy between SISH and FISH ratios.^27^ Another potential source of disagreement between ISH techniques is heterogeneity.^23^,^24^,^28^ Although heterogeneity occurred in up to 52% of our amplified cases, it did not cause the discrepancies, because, before scoring, screening of the entire slide took place.

Another interesting aspect to consider is the concordance of ratios between the different ISH techniques. In agreement with some authors^33^ and in disagreement with others,^25^,^26^ there seems to be, in our series, a weak trend towards lower ratios for FISH than for brightfield ISH. This should be taken into consideration if, in the future, the ratio has predictive value, as has been demonstrated in the neoadjuvant setting of breast carcinoma patients.^35^ Interestingly, the HERA trial has failed to confirm that the degree of *HER2* amplification influences the benefit from adjuvant trastuzumab. However, it is important to emphazise that central FISH analyses were only available in 61% of the patients randomized, and that the analyses were performed in two different laboratories.^36^ Following that line of reasoning, in the future, large trials could benefit from the availability of robust automated hybridization assays that allow for a rapid (<24 h), reliable and permanent ISH technique.

An analysis of the literature on ISH in GC is consistent with our results, although there are no data yet on the use of dual-colour brightfield ISH approaches. A recent publication reviewed this matter. An analysis, combining this information with our literature shows that, in 2513 samples, the mean *HER2* positivity rate was 16.5% (range 6.9–42.6%), very similar to the results of our series.^22^,^34^,^37^–^39^ Agreement exists that this alteration is associated with GCs of the intestinal type, but it is controversial whether it is homogeneous or focal.^39^,^40^ In the present study, *HER2* amplification was indeed more frequent in that histological type and heterogeneous in up to 52% of the samples. This result emphasizes the need to screen the whole slide before scoring, a post-analytical approach that is easier with SISH than with FISH.

The implementation of FISH in local laboratories has fallen below the initial expectations (approximately 4% of hospitals with <300 beds), according to a recent CAP survey.^41^ Therefore, it is likely that interpretation of gastric *HER2* SISH will be performed by pathologists who are not familiar with FISH. SISH has three main advantages over FISH (permanent record, brightfield post-analytical phase and fully automated analytical phase) that are particularly relevant in gastric *HER2* assessment: (i) SISH slides are easier to screen at low power (important with heterogeneity, as mentioned above); (ii) co-localization of ISH and IHC findings is straightforward with brightfield assays (approximately 8–13% of GCs may exhibit HER2 genetic–protein discordances);^2^ and (iii) the availability of a fully automated SISH method that allows for rapid subgroup analysis of the ToGA trial samples (mining of this database is of great interest with regard to understanding why patients who are IHC 0 or 1+ and FISH-positive do not seem to benefit from trastuzumab).^2^

In summary, we have compared two different ISH approaches for *HER2* testing in GC. The excellent concordance and the absence of false-negative cases validate this novel, automated, dual-colour SISH method. The few discrepant results with FISH were caused by polysomy. This shortcoming may be avoided by raising the cut-off for amplification (≥3). Consensus at global and national levels is urgently needed in this setting as a framework for analytical (technical) and post-analytical (interpretative) training.^42^ The implementation of new methodology should be based on GC data but take into serious consideration the previous vicissitudes in the experience of breast cancer HER2 testing.^43^

## Abbreviations

CAP: College of American Pathologists
Ch: cisplatin plus fluoropyrimidine
Ch+T: cisplatin plus fluoropyrimidine plus trastuzumab
DAPI: 4′,6-diamino-2-phenylindole
DNP: dinitrophenol
EGFR: epidermal growth factor receptor
FISH: fluorescence *in-situ* hybridization
GC: gastric carcinoma
HER2: human epidermal growth factor receptor 2
IHC: immunohistochemistry
ISH: brightfield *in-situ* hybridization
PCR: polymerase chain reaction
SISH: silver-enhanced *in-situ* hybridization
